# Fecal calprotectin measurement to detect recurrence of solitary juvenile polyps: A case report

**DOI:** 10.1097/MD.0000000000035448

**Published:** 2023-10-27

**Authors:** Maika Kudoh, Toshihiko Kakiuchi, Masato Yoshiura, Motohiro Esaki, Muneaki Matsuo

**Affiliations:** a Department of Pediatrics, Faculty of Medicine, Saga University, Saga, Japan; b Division of Gastroenterology, Department of Internal Medicine, Faculty of Medicine, Saga University, Saga, Japan.

**Keywords:** calprotectin, hemorrhage, pediatrics, polyps, recurrence

## Abstract

**Rationale:**

Juvenile polyps (JPs) are the most common polyp type and can be observed in 1% of all preschoolers. The peak incidence is observed at ages 3 to 5 years, constituting 90% of all polyps in children. Elevated levels of fecal calprotectin (FC) are often seen in children with JPs.

**Patient concerns:**

A 15-month-old girl was referred to our hospital for blood on the stool surface persisting for 3 months. She was healthy, with no abdominal pain, diarrhea, anorexia, or weight loss and no complaints other than hematochezia. Her physical examination, vital signs and laboratory date were unremarkable.

**Diagnosis:**

JPs

**Intervention:**

Total colonoscopy for her found 2 JPs in the sigmoid colon, which were subsequently resected endoscopically.

**Outcomes:**

At the age of 5 years, this patient again had bloody stools. Her FC measurement at that time was 1020 mg/kg, which normalized to 42 mg/kg 3 months after her second resection.

**Lessons:**

Single or multiple solitary JPs require follow-up that fully considers the possibility of recurrence. Establishing a method for early confirmation of JP recurrence based on bloody stools, fecal occult blood testing, and FC measurement is necessary.

## 1. Introduction

Juvenile polyps (JPs) were first described by Versé in 1908 and were considered malignant lesions.^[1]^ Later, however, Morson^[2]^ showed that JPs are benign hamartomas. In the absence of other lesions, isolated polyps are defined as solitary juvenile polyps (SJPs); they are of epithelial origin^[3]^ and result from structural rearrangement of the mucosa secondary to an inflammation. SJPs in the gastrointestinal tract are also called retention, inflammatory, and cystic polyps. They are the most common polyp types and can be observed in 1% of all preschoolers. The peak incidence is observed at ages 3 to 5 years, constituting 90% of all polyps in children. Although a single SJP is found in about half the cases, 2 to 10 polyps can be found in the other half.^[3]^ Mandhan^[4]^ reported that approximately 7% of JPs relapse within 1 year.

Calprotectin is a 36 kDa member of the S100 family of proteins. It is derived predominantly from neutrophils and has direct antimicrobial effects and a role within the innate immune response. Its concentration in feces is about 6 times that in plasma.^[5]^ The presence of calprotectin in feces is quantitatively related to neutrophil migration toward the gastrointestinal tract; thus, it represents a useful marker of intestinal inflammation.^[6]^ Mitorogiorgyou et al^[7]^ reported that fecal calprotectin (FC) is often elevated in children with JPs.

Here, we report a case of recurrent SJPs in which FC elevation was useful for diagnosis. Written informed consent for case report publication was obtained from this patient parents.

## 2. Case report

**Figure 1.**
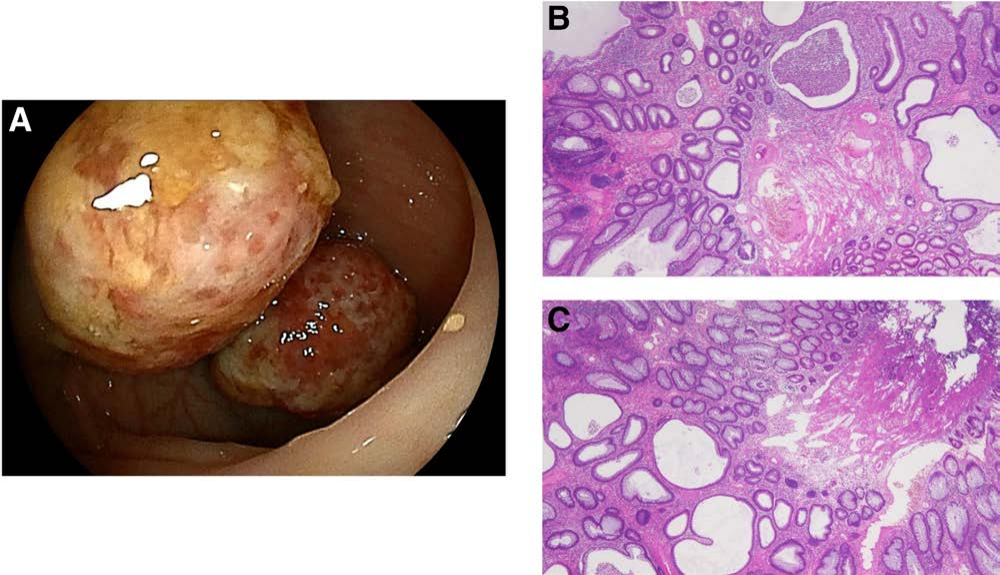
Endoscopic and pathology findings from polyps in a 15-mo-old patient. (A) Total colonoscopy revealed 2 polyps in close proximity to one another in the sigmoid colon. (B and C) Pathology findings reported variably dilated microcystic ducts and extensive edematous and inflammatory stroma. These 2 polyps were therefore diagnosed as juvenile polyps.

A 15-month-old girl was referred to our hospital for blood on the stool surface persisting for 3 months. She was healthy, with no abdominal pain, diarrhea, anorexia, or weight loss and no complaints other than hematochezia. Her physical examination and vital signs were unremarkable. Parameters from a complete blood count were white blood cells, 9900/mL (normal range [NR]: 7000–15,000/mL); hemoglobin, 12.4 g/dL (NR: 13.7–16.8 g/dL); and platelets, 415 × 10^3^/mL (NR: 158–348 × 10^3^/mL). Laboratory test findings included total protein, 6.2 g/dL (NR: 6.8–8.1 g/dL); albumin, 4.0 g/dL (NR: 4.1–5.1 g/dL); C-reactive protein, 0.01 mg/dL (NR: <0.14 mg/dL); serum amyloid A protein, 1.7 µg/mL (NR: <8.0 µg/mL); and erythrocyte sedimentation rate, 10 mm/hour (NR: <17 mm/hour). No infection was detected in stool culture tests. Total colonoscopy (TCS) was performed to determine the cause of the hematochezia, and 2 polyps were found in close proximity to one another in the sigmoid colon (Fig. 1A). No redness or aphthous ulceration of the intestinal mucosa suggestive of inflammatory bowel disease was observed. After other bleeding-causing factors had been ruled out, the 2 polyps were resected endoscopically. Pathology findings for the resected specimens reported variably dilated microcystic ducts and extensive edematous and inflammatory stroma (Fig. 1B and C). The patient had no family history of gastrointestinal polyposis. JPs were therefore diagnosed. No postoperative hematochezia was noted, and the patient medical follow-up was discontinued after 3 months.

**Figure 2.**
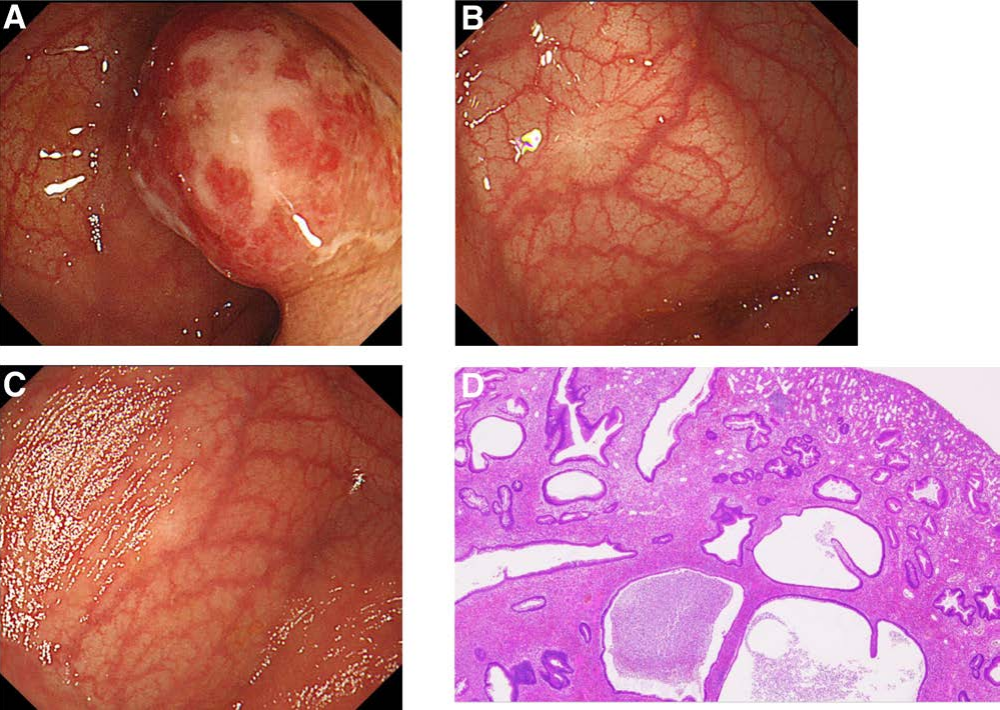
Endoscopic and pathology findings from a polyp in the same patient at 5 yr of age. (A) Total colonoscopy revealed a single polyp at the sigmoid colon that was located more in the oral direction than the previous 2 polyps had been. (B and C) Two scars were observed at the sites where the previous polyps had been excised. (D) Pathology findings reported variably dilated microcystic ducts and extensive edematous and inflammatory stroma. This polyp was therefore diagnosed as a recurrent juvenile polyp.

At the age of 5 years, this girl again had bloody stools. As in her first presentation, the patient was healthy, with no abdominal pain, diarrhea, anorexia, or weight loss and no complaints other than hematochezia. Her vital signs and physical examination showed no obvious abnormalities. Parameters from a complete blood count were white blood cells, 7900/mL; hemoglobin, 12.3 g/dL; and platelets, 373 × 10^3^/mL. Laboratory test findings included total protein, 6.5 g/dL; albumin, 4.0 g/dL; C-reactive protein, 0.09 mg/dL; serum amyloid A protein, 6.3 µg/mL; and erythrocyte sedimentation rate, 6 mm/hour. Her FC measurement at this time was 1020 mg/kg (NR: <50 mg/kg). No clinical signs or laboratory findings suggestive of inflammatory bowel disease other than hematochezia and elevated FC were evident. A second TCS revealed a single polyp at the sigmoid colon (Fig. 2A). Compared with the previous 2 polyps, this polyp was located more in the oral direction, and 2 scars were observed at the sites where the previous polyps had been excised (Fig. 2B and C). The location of the new polyp was thus clearly different from those of the previous polyps. The cause of the bloody stools in this second instance was diagnosed as a recurrent JP, which was resected endoscopically as before. Pathology findings for the newly resected specimen reported variably dilated microcystic ducts and extensive edematous and inflammatory stroma (Fig. 2D). Bloody stools did not recur after this resection, and the patient FC measurement normalized to 42 mg/kg 3 months afterward. Three months after the most recent polypectomy, this patient has so far experienced no recurrence of bloody stools.

## 3. Discussion

The clinical course in the present case suggests 2 important indications. First, single or multiple SJPs require follow-up that fully considers the possibility of recurrence. Second, FC might be useful as an indicator of JP recurrence.

Patients with fewer than 3 polyps have generally been considered to be at low risk for polyp recurrence; however, no evidence-based pediatric practice guidelines have yet emerged.^[8]^ Mandhan^[4]^ reported that approximately 7% of JPs recur within 1 year. Poddar et al^[9]^ reported that JPs recurred in 5% of children with such polyps. Recently, however, Fox et al^[10]^ reported that JPs recurred in 21 of 47 patients (44.7%) after initial eradication, including in 3 of 18 patients (16.7%) presenting with a single polyp—a much higher recurrence rate than previously reported, suggesting that children who present with even a single polyp might continue to form polyps over time. Because of a potential for neoplastic transformation, JPs should be removed even if asymptomatic.^[9]^ Thus, JP recurrences after resection must be diagnosed. In contrast, pediatric colonoscopy is not without risk: the complication rate can reach 1.1%, with a perforation rate in the 0.01% to 0.11% range.^[11]^ Because endoscopy in children is difficult and highly invasive, a method other than surveillance endoscopy is needed to predict JP recurrences.

The utility of elevated FC as a diagnostic aid has been reported in colonic JPs.^[12,13]^ JPs are composed of inflammatory cells, including many neutrophils, and the mucosal surface is often very friable. Exfoliation of cells into the stool would be expected to result in increased levels of fecal inflammatory markers.^[14]^ The report by Das et al that colonic JPs are associated with increased FC that normalizes after polypectomy^[12]^ is echoed in the present case, in which FC normalized after JP resection. FC measurement could therefore be recommended as a noninvasive screening biomarker (instead of frequent colonoscopies) to detect the presence of polyps and polyp recurrence.

Recently, ultrasonography has been used as a noninvasive method to detect colorectal polyps in pediatric patients. Hosokawa et al^[15]^ reported that the accuracy of ultrasonography in detecting colorectal polyps without any colon preparation was 89% (95% confidence interval, 80%–95%) in their cohort. Polyps in the rectum and relatively small polyps accounted for several false-negative results. Wei et al^[16]^ concluded that that ultrasonography is a valid, accurate, and safe technique for examining children for colorectal polyps. However, polyp detection by ultrasonography requires extensive experience and special techniques, which hinders the spread of ultrasonography for this purpose.

Based on our experience in the present case, we recommend these follow-up methods for JPs after first resection. For JPs in the left colon to the rectum, FC should be measured when bloody stool appears macroscopically; then, if FC is elevated, TCS should be performed, with inflammatory bowel disease being considered as a differential. For JPs in the oral side of the left colon, regular fecal occult blood tests should be performed. If occult blood is found, FC should be measured, and if FC is elevated, TCS should be performed. However, no evidence has yet been developed about how frequently to administer fecal occult blood tests or how to detect small intestinal JPs. Further investigation is therefore required.

To summarize, single or multiple SJPs require follow-up that fully considers the possibility of recurrence. Establishing a method for early confirmation of JP recurrence based on bloody stools, fecal occult blood testing, and FC measurement is necessary.

## Acknowledgments

The authors thank the patient family for providing consent and granting permission to draft and publish this case report.

## Author contributions

**Conceptualization:** Maika Kudoh, Toshihiko Kakiuchi.

**Data curation:** Maika Kudoh, Toshihiko Kakiuchi, Masato Yoshiura.

**Formal analysis:** Toshihiko Kakiuchi.

**Investigation:** Maika Kudoh, Toshihiko Kakiuchi.

**Methodology:** Maika Kudoh, Toshihiko Kakiuchi.

**Supervision:** Motohiro Esaki, Muneaki Matsuo.

**Validation:** Motohiro Esaki, Muneaki Matsuo.

**Writing – original draft:** Maika Kudoh, Toshihiko Kakiuchi.**Writing – review & editing:** Motohiro Esaki, Muneaki Matsuo.
